# Teratology

**Published:** 1877-11-20

**Authors:** D. Newcomer

**Affiliations:** Mt. Morris, Illinois


### TERATOLOGY.

BY D. NEWCOMER, M. D.

Mr. Editor.—In the September numbar of your journal, under the caption of "Maternal Impressions," Dr. George A. Dyer asks this question, "Did they so occur from impressions made on the mother?" I would answer, no. It is true, the cases adduced seem sufficiently striking. There is, however, numerous difficulties in the way of accepting the cause assigned. If a child is born with any malformation the recollection of the mother is racked to discover if something did not occur during gestation to which the appearance may be referred, and it is not often difficult to find some plausible means of explanation. I have had two cases occur in my own practice in which the malformation was similar, if not exactly like the first case described by Dr. Dyer. The first one, the mother (strange as it may seem), could give no cause for the malformation. The second one, after refreshing her memory, discovered that she had been badly frightened, while walking out with her husband, by treading on a toad, As soon as this discovery was made the good old ladies present saw, as a natural sequence, an exact likeness of the toad in the deformed child before them. Thus, by a little stretch of the imagination, it could be made to resemble either the "Wild Australian Children," or a toad, and, when we reflect that so large a number of women, sometime during pregnancy, have been the subjects of severe frights, fear or anxiety, and so few monstrosities born (about one in every three thousand), we must look elsewhere for the efficient cause of such freaks of nature. This, it may be said, is a sort of negative argument; it is, however, none the le s conclusive. The communication b$^{S}$tween the mother and *fœtns* is of the most indirect kind, the child can only be affected by the nutriment sent to it by the mother, which may be deficient in quantity or vitiated in quality. In either case the whole organism would be alike affected, and not only a particular part. Any violent mental emotion in the mother may thus destroy the life of the *fœtus* in *utero* by modifying the quantity or the quality of the nutritive matter sent to it. I cannot well conceive how any impression or influence on the imagination of the mother could suspend or arrest the development of organs in the *fœtus*, of which she knows little or nothing. We know that in the lower animals, and even in vegetable life, we have frequent cases of malformation, or "freaks of nature," in which maternal impressions cannot be invoked as the cause. In the case of the woman who saw, sometime during gestation, a man with one arm off, and her child being born with like defect, the arm must have been already formed, and the influence of the mother's "fancy" must have been exclusively exerted upon its absorbents, so as to cause them to take up again that which had already been deposited. It is difficult for us to believe, in this case, that the effect can, in any way, be connected with the assigned cause. It would be much easier to presume that the coincidence in such cases has been accidental. Cases of hair-lip are constantly occurring, yet we never, in such cases, have maternal impressions given as a cause.

M. Dareste has, by various experiments with incubation of eggs, succeeded in submitting to direct observation the evolution of most types of simple monstrosity, and he states, as one of the most general results obtained, "that monstrosities have always their origin in that period of embryonic life when the embryo is entirely formed of homogenious blastema. The monstrous organs first appear with all their teratological characters in blastema which has been previously modified."

In conclusion, M. Dareste remarks that, "though his teratogenic researches have been limited to a single species, they have a much more extensive reference. Indeed, the teretalogical types and processes of formation cited in birds are exactly the same as are observed in mammifers and in fishes * * * This identity of teratological types in mammifers, birds and fishes, is a necessary consequence of the unity of type in vertebrated animals."

From the experiments of M. Dareste it would seem that monstrosities are the result of an arrest of development at some period of embryonic ar fœtal life, and that such arrest cannot be caused by any impressions made upon the mind of the mother, but from causes inherent in the germ or embryo itself.

Mt. Morris, Illinois.

